# One Way to Achieve Germination: Common Molecular Mechanism Induced by Ethylene and After-Ripening in Sunflower Seeds

**DOI:** 10.3390/ijms19082464

**Published:** 2018-08-20

**Authors:** Qiong Xia, Marine Saux, Maharajah Ponnaiah, Françoise Gilard, François Perreau, Stéphanie Huguet, Sandrine Balzergue, Nicolas Langlade, Christophe Bailly, Patrice Meimoun, Françoise Corbineau, Hayat El-Maarouf-Bouteau

**Affiliations:** 1 Sorbonne Université, IBPS, CNRS, UMR 7622, 75005 Paris, France; xiaqiongupmc@gmail.com (Q.X.); marine.pellen@upmc.fr (M.S.); maha.ponnaiah@gmail.com (M.P.); christophe.bailly@upmc.fr (C.B.); patrice.meimoun@upmc.fr (P.M.); francoise.corbineau@upmc.fr (F.C.); 2 Plateforme Métabolisme-Métabolome, Institute of Plant Sciences Paris-Saclay (IPS2), UMR 9213/UMR1403, CNRS, INRA, Université Paris-Sud, Université d’Evry, Université Paris-Diderot, Sorbonne Paris-Cité, Saclay Plant Sciences, Bâtiment 630, 91405 Orsay, France; francoise.gilard@ips2.universite-paris-saclay.fr (F.G.); stephanie.huguet@ips2.universite-paris-saclay.fr (S.H.); 3 Institut Jean-Pierre Bourgin, INRA, AgroParisTech, CNRS, Université Paris-Saclay, 78000 Versailles, France; francois.perreau@versailles.inra.fr; 4 Unité de Recherche en Génomique Végétale (URGV), 91057 Evry CEDEX, France; sandrine.balzergue@inra.fr; 5 IRHS, équipe EPICENTER, 49071 Beaucouzé CEDEX, France; 6 LIPM, Université de Toulouse, INRA, CNRS, 31326 Castanet-Tolosan, France; nicolas.langlade@inra.fr

**Keywords:** seed, dormancy, hormones, transcriptomics, metabolomics

## Abstract

Dormancy is an adaptive trait that blocks seed germination until the environmental conditions become favorable for subsequent vegetative plant growth. Seed dormancy is defined as the inability to germinate in favorable conditions. Dormancy is alleviated during after-ripening, a dry storage period, during which dormant (D) seeds unable to germinate become non-dormant (ND), able to germinate in a wide range of environmental conditions. The treatment of dormant seeds with ethylene (D/ET) promotes seed germination, and abscisic acid (ABA) treatment reduces non-dormant (ND/ABA) seed germination in sunflowers (*Helianthus annuus*). Metabolomic and transcriptomic studies have been performed during imbibition to compare germinating seeds (ND and D/ET) and low-germinating seeds (D and ND/ABA). A PCA analysis of the metabolites content showed that imbibition did not trigger a significant change during the first hours (3 and 15 h). The metabolic changes associated with germination capacity occurred at 24 h and were related to hexoses, as their content was higher in ND and D/ET and was reduced by ABA treatment. At the transcriptional level, a large number of genes were altered oppositely in germinating, compared to the low-germinating seeds. The metabolomic and transcriptomic results were integrated in the interpretation of the processes involved in germination. Our results show that ethylene treatment triggers molecular changes comparable to that of after-ripening treatment, concerning sugar metabolism and ABA signaling inhibition.

## 1. Introduction

Seed dormancy is a fundamental adaptive trait that enables species persistence by blocking mature seed germination until conditions become favorable for seedlings establishment. Seed dormancy is alleviated during a period of dry storage, called after-ripening, after which dormant seeds are able to germinate. Germination begins with water uptake by the seed and ends with the start of the embryonic axis elongation. After-ripening depends on environmental factors such as temperature, light, oxygen, and moisture, which affect the seed hormonal balance determining the dormancy alleviation. The hormonal regulation of dormancy and germination is well established (for review [1]). Abscisic acid (ABA) is involved in the induction and the maintenance of seed dormancy, while other hormones such as gibberellic acid (GA), ethylene (ET), or brassinosteroids are positive regulators of seed germination [2]. The ethylene regulation of germination and dormancy has been demonstrated using physiological studies or mutants affected in ethylene synthesis and signaling. This regulation was related to the direct ethylene action, but also to ABA sensitivity change. In fact, ethylene overcomes the inhibitory action of ABA on germination in numerous species (for review [3]). In sunflowers, the ethylene supply breaks the dormancy and the endogenous ethylene is increased by dormancy alleviation treatments, with the addition of methylviologen, a reactive oxygen species (ROS)-generating compound, or cyanide (HCN) [4,5,6]. The after-ripening did not trigger an ethylene increase before the end of germination sensu stricto in sunflowers [6]. It was shown that a peak of ethylene emission was concomitant with radicle protrusion through the seed coat in different species (reviewed in [3]). However, the ethylene responsive factor (ERF1) gene expression was five-fold higher in the non-dormant rather than in the dormant embryos, and was markedly stimulated by HCN in sunflowers [4], suggesting that ethylene signaling acts before the peak recorded during radicle protrusion, probably due to ethylene production below the detectable level. Hormonal regulation is integrated in the seed response to environmental conditions, such as temperature [7,8,9] or nitrate [10].

Various global analyses have been used in the last years in order to discover and connect the pathways involved in the dormancy alleviation process. Transcriptional studies showed that the genes related to ABA and stress response, such as late embryogenesis or heat-shock proteins, are over-represented in dormant (D) seeds, while the genes associated with protein synthesis, including RNA translation, protein degradation, and also cell wall modification, were reduced compared to the after-ripened, non-dormant (ND) seeds [11,12,13]. The same protein families have been identified in the proteomic analyses of seed dormancy. Stress-related proteins were more abundant in D seeds, while the proteins related to the protein metabolism and folding, energy, and glycolysis are up-regulated in germinating seeds [9,14,15]. Gao et al. [16] showed in an integrated study of wheat seed proteome and mRNA oxidation, that after-ripening is mediated by the differential expression of the proteins involved in proteolysis, cellular signaling, energy, and translation. Translation is one of the multilayers of processes that regulate the dormancy alleviation process by mRNA selection for the recruitment to polysomes [17,18]. As the last layer, metabolomic studies bring information about metabolites, which are the integration of genomic, transcriptomic, and proteomic to cellular biochemistry and metabolism [19]. Fait et al. [20] showed that TCA-cycle intermediates, 2-oxoglutarate and isocitrate, as well as dehydroascorbate, 3-phosphoglycerate, Fructose 6P, and Glucose 6P, increased, while numerous amino acids and sugars were reduced during the Arabidopsis seed vernalization and germination sensu stricto. The comparison of two wheat cultivars with a contrasting dormancy status showed that fatty acids, oxalate, hormones, the raffinose family of oligosaccharides, and amino acids are key metabolic pathways [21]. Recently, the central metabolism has been shown to be fine-tuned in dormancy control [9]. Although these omics results enrich the network scheme of the dormancy process, there is very limited data on hormone treatment and ethylene in particular.

Thus, our aim in this study was to unravel the network between hormones, ABA and ET, and seed metabolism in the control of sunflower seed dormancy and germination. We performed transcriptomic and metabolomic analyses during imbibition comparing germinating seeds, ND and D treated with ethylene (D/ET), and low-germinating seeds D and ND treated with ABA (ND/ABA). Our data highlight a high similarity between ethylene and after-ripening treatments as well as a determinant cross-talk with ABA signaling in the induction of germination.

## 2. Results

### 2.1. Seed Physiology

**Figure 1:**
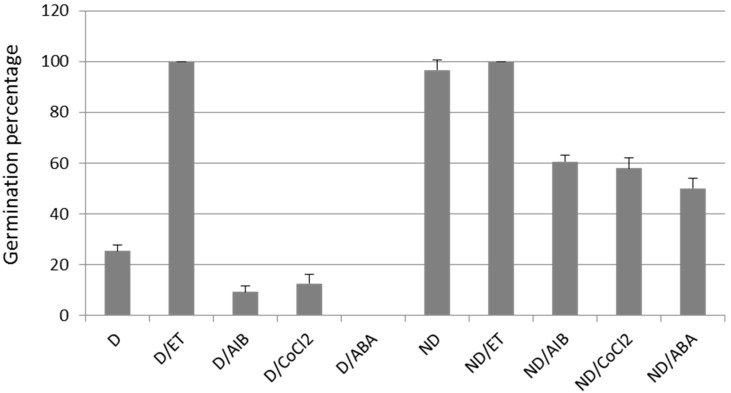
Germination percentage of sunflower dormant and non-dormant embryos after seven days of imbibition at 10 °C on water, dormant (D), and non-dormant (ND); or with ethylene (ET), D/ET and ND/ET; or ethylene inhibitors, α-aminoisobutyric acid (AIB) and cobalt chloride (CoCl_2_); or with abscisic acid (ABA), D/ABA, and ND/ABA. Values are means of three replicates of 50 embryos ± standard deviation.

Figure 1 shows that sunflower seeds are deeply dormant at harvest, as only 25% of seeds can germinate after seven days of imbibition at 10 °C. After-ripening alleviates this dormancy as ND seeds germinate at 97% (Figure 1). Ethylene treatment is efficient in dormancy breaking; it allows for the germination of D seeds at 100% (Figure 1). The proportion of seeds that germinate in D or ND was halved by the ethylene synthesis inhibitors, α-aminoisobutyric acid (AIB) and cobalt chloride (CoCl_2_), and the ABA treatments. AIB and CoCl_2_ inhibit 1-aminocyclopropane 1-carboxylic acid (ACC)-oxidase, the enzyme that produces ethylene from the oxidation of ACC [22]. These results suggest that the ethylene synthesis is important for sunflower seed germination to counteract ABA-induced dormancy (Figure 1).

**Figure 2:**
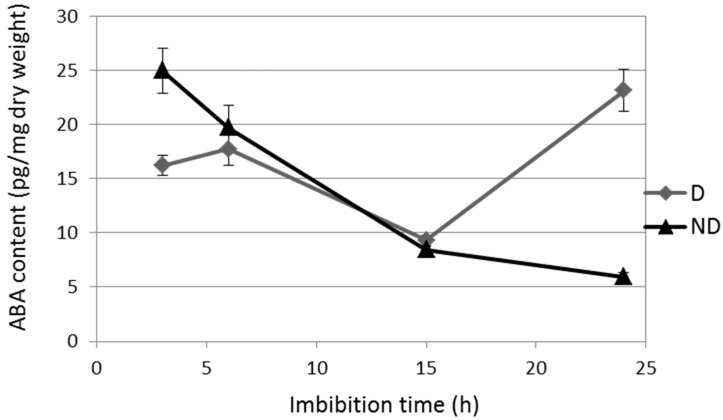
ABA content in the embryonic axis of dormant (D) and non-dormant (ND) during imbibition at 10 °C. Values are means of four replicates ± standard deviation.

With ABA being responsible for dormancy maintenance during imbibition, the ABA content has been quantified in order to characterize the kinetics of dormancy breaking during the chosen imbibition times. Figure 2 shows that in D seeds, the ABA content is reduced at 15 h and then increased at 24 h of imbibition, while in ND seeds, the ABA decreased continuously during imbibition. The largest variation between D and ND seeds occurred thus at 24 h of imbibition (Figure 2).

### 2.2. Metabolome Analysis

**Figure 3:**
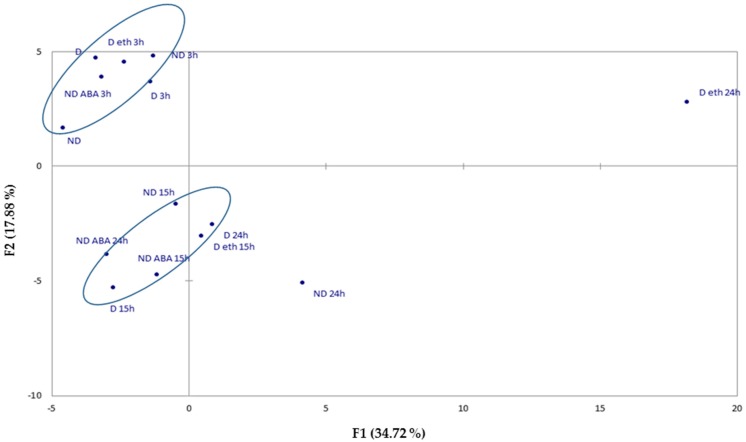
PCA analysis of metabolite content in dry dormant (D), dry non-dormant (ND), and D imbibed on water for 3 h (D 3 h), 15 h (D 15 h), 24 h (D 24 h); ND imbibed on water for 3 h (ND 3 h), 15 h (ND 3 h), and 24 h (ND 24 h); D treated with ethylene for 3 h (D/eth 3 h), 15 h (D/eth 15 h), and 24 h (D/eth 24 h); and ND treated with ABA for 3 h (ND/ABA 3 h), 15 h (ND/ABA 15 h), and 24 h (ND/ABA 24 h). Results are the means of three biological repetitions.

A non-targeted metabolome analysis was performed on D and ND dry seeds; D and ND seeds imbibed on water; and ethylene-treated D (D/ET) and ABA-treated ND (ND/ABA) seeds for 3, 15, and 24 h at 10 °C. A PCA analysis using the Pearson correlation (*p* < 0.005) of the metabolites content showed sample differentiation in three groups (Figure 3 and Table S1). One group contains dry D and ND seeds in addition to imbibed D and ND seeds on water, D/ET, and ND/ABA for 3 h; demonstrating that no significant change in metabolites happened in the first three hours of imbibition. A second group contains imbibed seeds for 15 h for all treatments, but also D imbibed on water and ND/ABA at 24 h, indicating that the metabolite profile at 15 h cannot discriminate between germinating and low-germinating seeds, and that the metabolism of low-germinating seeds (D and ND/ABA) did not change between 15 and 24 h. Interestingly, only ND and D/ET for 24 h are represented in the last group, suggesting that the differentiation between germinating and low-germinating seeds occurred between 15 and 24 h of imbibition. Interestingly, the ABA treatment on ND seeds maintained their metabolism at 24 h, close to that of the 15 h group with D 24 h (Figure 3). Thus, based on this analysis, we can conclude that imbibition has an influence on the change of the entire metabolism between 3 and 15 h, whatever the physiological state, but that metabolic changes related to germination potential occur at 24 h. Therefore, altered metabolites were analyzed at 24 h of imbibition comparing ND and D/ET to D, as well as ND/ABA to ND seeds.

**Figure 4:**
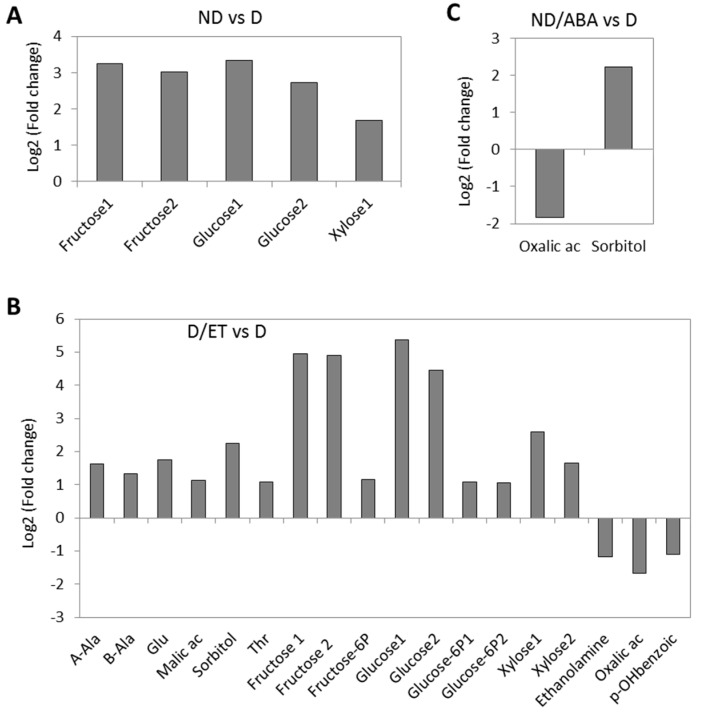
Main relative metabolite content change expressed as log2 (fold) change of (**A**) non-dormant compared to dormant seeds (ND vs. D); (**B**) dormant treated with ethylene compared to dormant (D/ET vs. D); (**C**) non-dormant treated with ABA compared to dormant (ND/ABA vs. D). A-Ala—α-alanine; B-Ala—β-Alanine; Glu—glutamine; Thr—Threonine; p-OHbenzoic—p-hydroxybenzoic acid.

It is worth noting that among the 86 metabolites detected (Table S1), significant changes between the different conditions (>2-fold at *p* >0.05) concerned only a few metabolites. In fact, only the glucose, fructose, and xylose contents changed between D and ND metabolite profiles (Figure 4A). They were highly accumulated in ND and D/ET. A change in glucose and fructose 6P was also noted in D/ET. The ABA treatment of the ND seeds did not allow for sugar accumulation as their content was comparable to that of D (Figure 4C). Only the oxalic acid and sorbitol contents change between ND/ABA and D. This change is comparable to that induced by the ET treatment (Figure 4B). A slight accumulation of some amino acids was observed in the ethylene treatment (Figure 4B). These results showed that the hexose content seems to be associated with the germination potential.

### 2.3. Transcriptome Analysis

**Figure 5:**
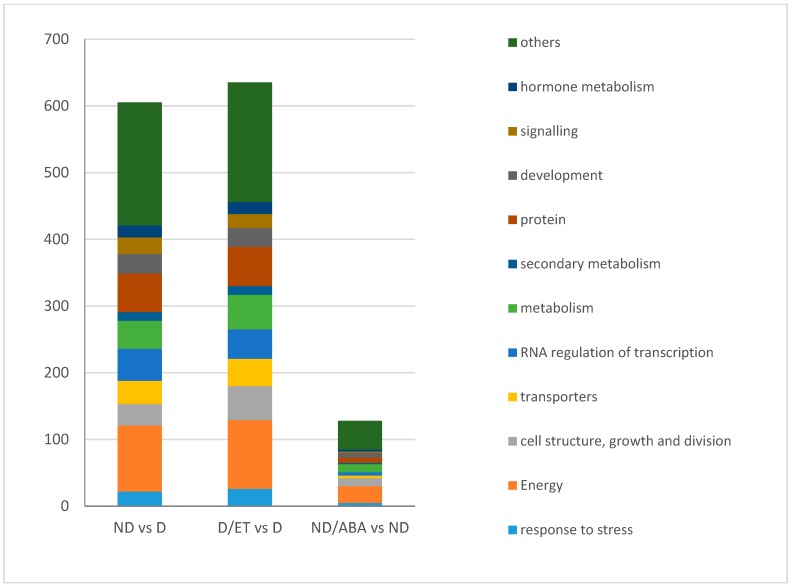
Gene ontology (GO) classification based on MapMan software and Ashburner et al. [23] of the genes altered in non-dormant compared to dormant seeds (ND vs. D); dormant treated with ethylene compared to dormant (D/ET vs. D); and non-dormant treated with ABA compared to non-dormant (ND/ABA vs. ND).

The transcriptomic change between the germinating and low-germinating seeds was studied by microarray analysis using the same conditions as in the metabolomic analysis (D, ND, D/ET, and ND/ABA at 24 h). Gene ontology (GO) annotations based on MapMan software and Ashburner et al. [23] showed that the altered transcripts in all of the treatments belong to the same classes (Figure 5).

**Figure 6:**
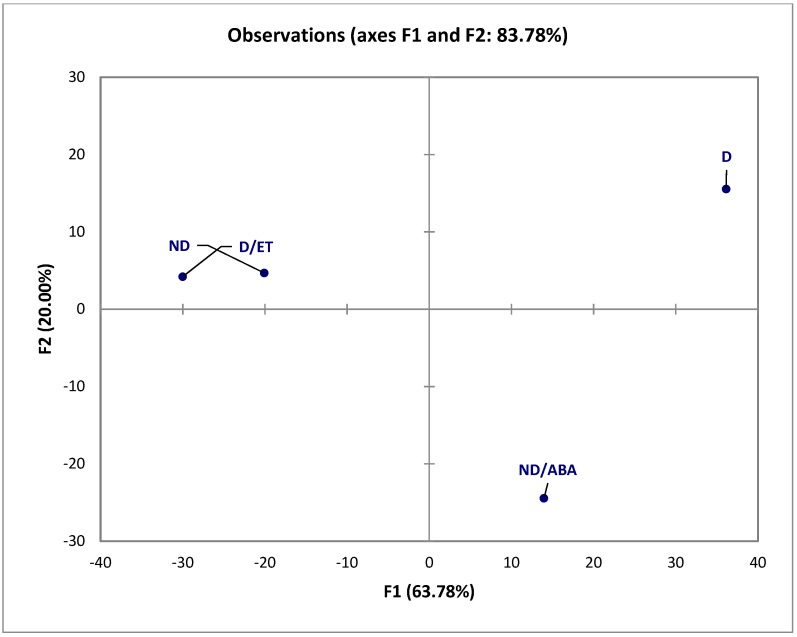
PCA analysis of transcriptomic change of dormant (D) imbibed on water and in the presence of ethylene (D/ET), and non-dormant (ND) imbibed on water or on abscisic acid ((ABA) ND/ABA) during 24 h at 10 °C.

The transcriptome analysis using PCA confirmed that in ND and D/ET, the gene variation is similar, at a high extent, to PCA axes F1 and F2, and can explain the variability at the score of 83.78% (Figure 6). The ABA treatment on ND seeds (ND/ABA) induced an important change in the gene expression compared to ND, but gets closer to D, especially on the major axis (*X* axis of 63.78%, Figure 6).

Interestingly, all of the altered genes in ND/ABA compared to D were altered oppositely in ND and D/ET, thus they could represent good candidates for germination induction (Table 1). Up-regulated genes in the germinating seeds and down-regulated genes in the low-germinating seeds are members of a sugar and cell wall metabolism, such as glycosyltransferase, β-xylosidase, or xyloglucan endotransglucosylase/hydrolase. Hormone metabolism-related genes are also represented by gibberellin-regulated (GAST1) protein homolog, *S*-adenosylmethionine decarboxylase, myeloblastosis (Myb)-related transcription factor family, or an APETALA2/ethylene-responsive element binding proteins (AP2/EREBP) member and *B12D*. The genes involved in signaling, such as mitogen activated protein kinase (MAPKKK) 20 and amino acid transport, or in development, such as pentatricopeptide and domains of unknown function (DUF) 3049, are also of high interest.

The genes regulated in the opposite way (down in germinating seeds and up in low-germinating seeds) are represented by members of lipid, cell wall, oxidation–reduction metabolism, late embryogenesis abundant (LEA) protein family, and also hormones metabolism regulators (Table 2). Nine genes belonging to the two categories have been used for qPCR validation (Figure S1). The microarray and qPCR values correlated at a high coefficient of 0.83.

The first version of the sunflower genome annotation (https://www.heliagene.org/) allowed us to analyze the cis-elements present in the 500 bp promoter regions of these common genes. Thirty-six percent (8 out of 22 sequences) of the up-regulated genes in ND and D/ET, and down-regulated in ND/ABA, contained a homeodomain binding site, ATHB6, a target of ABI1 and a negative regulator of ABA signaling [24]. Sixteen percent (4 of 24 sequences) of the down-regulated genes in ND and D/ET, and up-regulated in ND/ABA, contained a Z-box motif [25]. Z-box is recognized by the AtMYC2 and AtMYB2 binding factors shown to be transcriptional activators in ABA-inducible gene expression under drought stress in plants [26], and as a negative regulator of the JA/ET-dependent anti-fungal defense program [27]. These results confirm that ethylene and ABA cross-talk in germination execution in sunflower seeds.

## 3. Discussion

Sunflower seeds are dormant at harvest, and the exogenous ethylene application efficiently improved their germination (Figure 1). The involvement of endogenous ethylene in the regulation of sunflower seed dormancy and germination has been assessed by using inhibitors of ethylene synthesis. Figure 1 showed that the ethylene inhibitors reduced germination of the ND seeds, which clearly demonstrates that the ethylene evolved by the seeds plays an important role in breaking the embryo dormancy. Ethylene is important in germination promotion in several species [28]. Other hormones such as GA, auxin, or brassinosteroids, or small molecules like ROS and NO, can break dormancy depending on the species [2]. The dormancy release process may be specific to each species because of the seed structure or composition, environment, or crop selection, whereas, the dormancy induction and maintenance is common, as ABA has a conserved role in these processes across species [28,29]. Therefore, it is important when studying the dormancy related mechanisms to characterize the seed physiological state by quantifying the ABA during the imbibition times, as dormancy depends on the seed lots and environmental conditions related to the ABA content [30]. In sunflower seeds, the ABA content decreased similarly in the D and ND seeds upon imbibition until 15 h, and then it increased only in the D seeds at 24 h (Figure 2). This pattern has been observed in several species because of the regulation of both the ABA catabolism and neo-synthesis (for review [31]). The 24 h imbibition was also the time that allows for the differentiation between the metabolism of the germinating seeds (D/ET and ND) and the low-germinating seeds (D and ND/ABA). Furthermore, the ABA treatment on the ND seeds maintained their metabolism close to that of the D seeds (Figure 3). ABA seems to represent the key regulator of metabolism remodeling, in order to determine the seed germination capacity.

The metabolites comparison at 24 h of imbibition showed that only fructose, glucose, and xylose increased in the ND seeds when compared to D. They also increased in D/ET and decreased upon ABA treatment, as the ND/ABA hexoses content is similar to that of D (Figure 4 and Table S1). Glucose and fructose are the products of sucrose degradation. In our samples, the sucrose content did not change significantly between D and ND, or D/ET (Table S1). It can be explained by an alteration in both the sucrose generation and degradation processes. The sucrose synthase 3 (SUS3) gene expression is down-regulated in the germinating seeds, while it was induced in the ABA treated seeds (Table 2). Sucrose synthase catalyzes the reversible cleavage of sucrose into uridine diphosphate (UDP)-glucose and fructose. SUS is supposed to be important in the rate of seed development by the influence of the sucrose/hexose ratio on the pattern of the storage compounds in Arabidopsis [32]. The AtSUS3 transcripts were induced in mid seed development, increased in abundance in the mature seeds, and decreased to undetectable levels in the germinating seeds in Arabidopsis [33]. Our results suggest that the sucrose/hexose ratio may be determinant in the metabolism of seed germination.

Hexoses can also be generated by cellulose and hemicellulose depolymerization. In accordance, the β-xylosidase 2 and 4 transcripts were shown to be up-regulated in ND and D/ET, and down-regulated in ND/ABA (Table 1); they are involved in the conversion of (1–4)-β-d-xylan oligosaccharide to β-d-xylopyranose, which generates D-xylulose. Furthermore, xyloglucan endotransglucosylase/hydrolase (XTH), which cleaves and relegates the xyloglucan polymers is also up-regulated in ND and D/ET (Table S2). These proteins modify the cell wall to make it more extensible, thereby facilitating the cellular expansion [34]. XTH and xylosidase are induced by GA and ethylene, respectively [35,36]. In D/ET, the glucose 6P and fructose 6P were significantly over-accumulated (Figure 4), suggesting the activation of glycolysis. TCA is also activated by ethylene, as the oxalate content decreased and that of the malate increased (Figure 4). This result is in accordance with our previous work on the regulation of central metabolism during germination sensu stricto in dormancy control by temperature in sunflower seeds [9]. The accumulation of hexose phosphates and the TCA cycle intermediates has been reported during Arabidopsis seed stratification, which allows for dormancy breaking [20]. These results indicate that hormones cross-talk with the central metabolism in the control of dormancy alleviation and germination.

A PCA analysis of altered gene expression showed that ND and D/ET are highly similar (Figure 6). ND/ABA is however distinct from D, whereas their metabolism was comparable at 24 h (Figure 3 and Figure 4). Previous works argued that ABA treated seeds are not close to the dormant ones at the metabolic and physiological level [13,37,38]. In sunflowers, the exogenous application of ABA can inhibit germination but not efficiently in all seeds, as germination the percentage of ND/ABA remains two-fold higher than that of D (Figure 1). The ND/ABA seed population is therefore enriched by germinating seeds. However, we show here that a significant proportion of genes were altered oppositely in ND and D/ET compared to ND/ABA (Table 1). They correspond to almost all of the genes altered in the ND/ABA condition (Table 1 and Table S2). In addition to the sugar and cell wall-related genes discussed in the metabolic part, the genes related to hormone response were represented as up-regulated in germinating seeds, and down-regulated in low-germinating seeds, such as the gibberellin-regulated protein GASA4, which promotes GA responses and exhibits redox activity; *S*-adenosylmethionine decarboxylases (SAMDCs) involved in the polyamine biosynthetic pathway; Myb and AP2/EREBP transcription factors (TFs); and MAPKKK20 (Table 1). Some Myb TFs might function as key mediators of stress responses and can be regulated by hormones, such as ABA and JA [39,40,41]. It has been shown that a Myb TF, DUO1, is targeted by the MKKK20 pathway [42]. The AP2/EREBP transcription factors have been implicated in hormone, sugar, and redox signaling in the context of abiotic stresses, such as cold and drought [43]. RAP2.6, an *Arabidopsis* AP2/ERF family member, can function in multiple abiotic stresses during seed germination, including salt, osmotic, and cold stress [44]. On the other hand, ABA-responsive genes such as ABI2 and ABI5 binding protein 3, and stress-related genes (dehydrin and LEA) were represented in the down-regulated genes in the germinating seeds that are up-regulated in low-germinating seeds (Table 2). These elements are likely to be the target of a hormone cross-talk signaling pathway.

The genes involved in organelle functioning have been identified in up-regulated genes in germinating seeds, such as Seed Imbibition 1 (SIP1), B12D, and pentapeptide. SIP1.1 and SIP1.2 may function as water channels in the ER [45]. B12D1 was identified in the mitochondrion in rice seeds and was proposed to enhance the electron transport through mediating the iron and oxygen availability under flooded conditions during germination [46]. Pentapeptide is targeted to mitochondria or chloroplasts, binds one or several organellar transcripts, and influences their expression by altering the RNA sequence, turnover, processing, or translation. Their combined action has profound effects on organelle biogenesis and function, and, consequently, on photosynthesis, respiration, plant development, and environmental responses (for review [47]). These genes may be involved in the organelles functioning resumption necessary for germination.

Cytochrome P450 and glutathione peroxidase (GPX) were characterized in the down-regulated genes in the germinating seeds that are up-regulated in the low-germinating seeds. GPXs catalyze the reduction of lipid peroxides and other organic peroxides to the corresponding alcohols using thioredoxins as preferred electron donors [48,49]. The major function of GPXs in plants is the scavenging of phospholipid hydroperoxides, and thereby the protection of cell membranes from peroxidative damage [50]. On the other hand, recent data emphasized the role of GPXs in redox signal transduction under stressful conditions [51]. Redox-responsive genes such as RD21 and Pap15, which are up-regulated in D/ET, have been associated with sunflower seed dormancy alleviation induced by methyl viologen (MV), a ROS generating compound [6]. Similarly, the ABA signaling elements were down-regulated by MV, suggesting a cross-talk between the ROS and hormones in sunflower seed germination [6]. The common underlying mechanism may be redox signaling, as several redox genes were altered in ND and D/ET (e.g., glutathione S-transferase, which represent an important component of the redox system) (Table S2). The cross-talk between the ROS and hormones regulates many metabolic and developmental processes in plants (for review [52]). In the event that the germination is a conserved mechanism, after-ripening may trigger a redox potential change following the ROS amplification that determines the hormone signaling. Ethylene treatment may induce a redox potential change by its action on ROS production, evidenced in El-Maarouf-Bouteau et al. [6], as well as cross-talks with ABA signaling towards germination processes by reducing the sucrose/hexose ratio, which activates the TCA cycle, glycolysis, and organellar function.

This study evidenced that the ethylene treatment triggers comparable molecular change to the after-ripening treatment, suggesting that the dormancy alleviation is based on a unique execution program. Moreover, the ABA treatment induces the opposite regulation of genes and metabolites compared to ND and D/ET. It further provides a strong indication that the sucrose/hexoses ratio could be determinant in cell metabolism activation towards radicle elongation. Furthermore, transcriptomic data provides accurate information about the ABA and ethylene responsive genes in seeds, which will help to unravel the signaling network as a whole.

## 4. Materials and Methods

### 4.1. Seeds and Treatments

Sunflower seeds (*Helianthus annuus*) cultivar LG5665 were grown by Valgrain in an open field in the South of France in 2010. The mature seeds were stored at −20 °C to preserve dormancy (dormant seeds (D)), and at 20 °C for two months to release dormancy (non-dormant seeds (ND)). Treatment with ethylene was carried out by placing the D embryos on the water in the presence of 100 ppm of gaseous ethylene in closed Petri dishes, as described by Oracz et al. [4] (D/ET). The ABA and GA treatment consisted of ND seed imbibition with an ABA solution at 10^−5^ M (ND/ABA), and a GA solution at 10^−4^ M. The ethylene synthesis inhibitors treatment was done by imbibing the seeds with 1 mM of solution of inhibitors of ACC oxidase activity, α-aminoisobutyric acid (AIB), and cobalt chloride (CoCl_2_). All of the treatments were performed on embryos (seeds without pericarp) to focus on embryo dormancy. The embryonic axes [4] were isolated from the dry or imbibed seeds, frozen in liquid nitrogen, and stored at −80 °C until use for ABA quantification and omics analysis. The imbibition times were chosen in order to be prior to the end of germination *sensu stricto* (between 0 to 24 h) to target the processes that induce dormancy alleviation and not elongation and growth, which happened at 48 h of imbibition.

### 4.2. Germination Tests

Germination assays were carried out on 25 sunflower embryos (four replicates) in 9 cm Petri dishes on water or with various solutions in the darkness at 10 °C. The germination was counted daily and an embryo was considered as germinated when the radicle starts to elongate.

### 4.3. ABA Quantification

100 mg of freeze-dried axes were ground in 7 or 1.5 mL of extraction solvent, respectively (acetone:water:acetic acid, 80:19:1, *v*:*v*:*v*) containing 50 ng of deuterated ABA (purchased from Irina Zaharia, Plant Biotechnology Institute, National Research Council, Saskatoon, SSK, Canada, http://www.nrc-cnrc.gc.ca) as the internal standard. The samples were analyzed, as previously described [53]. The ABA was quantified using an LC-ESI-MS-MS system (Quattro LC; Waters, Manchester, UK, http://www.waters.com).

### 4.4. Metabolite Extraction, Identification, and GC-TOF Analyses

The metabolites were extracted from lyophilized samples using a methanol/water mixture (80/20 *v*/*v*). Metabolite derivatization, GC (gas chromatography)-TOF (time-offlight)-MS, data processing, and profile analyses were performed, as described in Su et al. [54]. Each metabolite was identified by comparison of the spectra with those that were obtained using the corresponding pure metabolite and its relative amount was calculated on the basis of the corresponding peak area compared with an internal standard (ribitol). The analysis was performed using a LECO Pegasus III with an Agilent (Massy, France) 6890N GC system and an Agilent 7683 automatic liquid sampler. The column was an RTX-5 w/integra-Guard (30 m (0.25 mm internal diameter) + 10 m integrated guard column) (Restek, Evry, France). The combination of the chromatography retention time (t) and parent mass pattern was used to selectively monitor the different metabolites. The different samples were spiked with an internal standard molecule (ribitol) to evaluate the level of analysis reproducibility. Of the metabolites, 86 among 144 (listed in Table S2) from the different samples were quantified by reporting the MS peak areas to the calibration curves, made with standard molecules of lactate, fructose, and urea at different concentrations.

### 4.5. RNA Extraction, Microarray Hybridization, and Analysis

The total RNA was extracted as previously described by Oracz et al. [5]. The RNA hybridization (two biological and two technical repetitions) was performed using Affymetrix Sunflower Gene WT Chip at the Affymetrix platform (INRA-URGV) and the protocol developed on sunflower seeds [6,55,56]. The statistical analyses were based on the *t*-test adjusted with the Bonferroni method [55,56]. A Bonferroni *p*-value less than 0.05 was required for a significant variation in the gene expression. The data are available on the gene expression omnibus (GEO) repository of the national center for biotechnology information (NCBI) at the accession number of GSE44165.

### 4.6. Real-Time Quantitative RT-PCR

The quantitative PCR was performed according to Meimoun et al. [56]. The relative expression was calculated according to Hellemans et al. [57], with HaEF1, Haβ-tubulin, and HaS19 as reference genes [6,56]. The gene names and corresponding primers used are listed in Supplemental Table S3. An arbitrary value of 100 was assigned to the dormant seed samples, which were used as control samples for normalization [58]. The results presented are the means ± standard deviation (SD) of three biological replicates.

## Figures and Tables

**Table 1:** MapMan classification (http://bar.utoronto.ca) of sunflower genes up-regulated in germinating, non-dormant (ND) and dormant (D)/ethylene (ET) compared to dormant (D), and down-regulated in low-germinating, ND/abscisic acid (ABA) compared to ND.

Gene IDs (https://www.heliagene.org/)	Homologue (TAIR or GeneBank Accession Number)	Description	Log2 Ratio ND vs. D	Log2 Ratio D/Et vs. D	Log2 Ratio ND/ABA vs. ND
**Cell Wall**					
Heli005917_st/HuCL00926C002/HanXRQChr02g0055461	AT2G28110	Exostosin family protein	1.65	2.72	−1.73
Heli024944_st/HuCL09512C001/HanXRQChr10g0285351	AT1G02640	beta-xylosidase 2 (BXL2)	2.15	3.29	−2.52
Heli030585_st/HuCL11891C001/HanXRQChr10g0293191	AT5G64570	beta-d-xylosidase 4 (XYL4)	1.49	2.43	−2.00
Heli009082_st/HuCL12520C001/HanXRQChr12g0371801	AT5G63180	pectate lyases and polygalacturonases	1.42	1.42	−1.95
Heli054183_x_st/HuCL00144C005	AT4G03210	(XTHs) xyloglucan endotransglucosylase/hydrolases	2.87	4.06	−2.65
**Cell**					
Heli006622_st/HuCL00145C001/HanXRQChr12g0376601	AT1G50010	tubulin alpha-2 chain (TUA2)	1.49	2.31	−1.59
Heli059476_st/HuCL06382C001/HanXRQChr15g0496411	AT1G74790	carbohydrate metabolic process	1.54	1.70	−1.99
**Secondary Metabolism**					
Heli000031_st/HuCL01414C001/HanXRQChr03g0080121	AT4G15560	Deoxyxylulose-5-phosphate synthase	2.10	1.63	−1.70
Heli006291_st/HuCL12958C001/HanXRQChr16g0522861	AT1G18590	sulfotransferase 17 (SOT17)	1.63	1.87	−1.72
**Hormone Metabolism**					
Heli001834_st/HuCL04379C001/HanXRQChr07g0199351	AT5G15230	GAST1 protein homolog 4	2.22	2.88	−2.50
**Polyamine Metabolism**					
Heli007531_st/HuCL00106C002/HanXRQChr12g0365201	AT3G02470	S-adenosylmethionine decarboxylase (SAMDC)	2.17	2.20	−2.03
**Minor CHO Metabolism**					
Heli002812_st/HuCL03745C001/HanXRQChr05g0160181	AT1G55740	seed imbibition 1 (SIP1)	2.12	1.46	−1.79
**RNA Regulation of Transcription**					
Heli012022_st/HuCL08506C001/HanXRQChr01g0020591	AT2G38090	Myb-related transcription factor family	2.50	2.38	−2.23
**Protein Postranslational Modification**					
Heli094295_st/HuAJ827835/	AT3G50310	mitogen-activated protein kinase kinase kinase 20 (MAPKKK20)	2.53	2.17	−2.44
**Development**					
Heli028832_st/HuCL10045C001/HanXRQChr12g0385621	AT3G48140	*B12D*	1.40	1.89	−1.85
Heli022386_st/HuCL11611C001/HanXRQChr11g0323801	AT4G36920	Integrase-type DNA-binding superfamily protein	1.87	2.19	−1.57
**Oxidation-Reduction**					
Heli032638_st/HuCL03867C002/HanXRQChr11g0339341	AGK88243.1	polyphenol oxidase	1.65	3.13	−2.82
**Transport**					
Heli033065_st/HuCL07407C001/HanXRQChr14g0456651	AT2G39130	Amino acid transporter	2.16	2.23	−1.78
**Not Assigned**					
Heli000358_st/HuCL03981C001/HanXRQChr11g0332871	AT1G22370	UDP-glucosyl transferase 85A5 (UGT85A5)	1.15	2.02	−1.62
Heli068232_x_st/HuBU031493/HanXRQChr10g0315221	AT3G02650	pentatricopeptide (PPR) repeat-containing protein	1.28	2.69	−1.81
Heli068743_x_st/HuBU030210/HanXRQChr10g0315221	AT3G02650	pentatricopeptide (PPR) repeat-containing protein	2.06	3.24	−2.37
Heli027060_st/HuDY915375/HanXRQChr17g0555081	AT1G53035	Protein of unknown function	1.84	2.36	−1.84
Heli029666_st/HuBU027813/HanXRQChr10g0291611	AT4G02810	Protein of unknown function DUF3049	2.21	2.11	−2.42
Heli066534_st/HuBU024903/HanXRQChr15g0492241	AT5G37010	Protein of unknown function	1.87	2.71	−1.75

**Table 2:** MapMan classification (http://bar.utoronto.ca) of sunflower genes down-regulated in germinating seeds, ND and D/ET, and up-regulated in low-germinating seeds, ND/ABA.

Gene IDs (https://www.heliagene.org/)	Homologue (TAIR or GeneBank Accession Number)	Description	Log2 Ratio ND vs. D	Log2 Ratio D/Et vs. D	Log2 Ratio ND/ABA vs. ND
**Lipid Metabolism**					
Heli009291_st/HuCL11228C001/HanXRQChr04g0124391	AT1G36160	acetyl-CoA carboxylase 1	−2.56	−2.84	1.88
Heli072022_st/HuBQ911641/HanXRQChr04g0124381	AT1G36160	acetyl-CoA carboxylase 1	−2.29	−1.32	2.10
Heli015269_st/HuBU027455/HanXRQChr14g0450531	AT3G27660	oleosin 4	−1.27	−1.40	1.59
**Hormone Metabolism and Signalling**					
Heli054510_st/HuDY929804/HanXRQChr09g0238691	AT5G57050	Protein phosphatase 2C family protein (ABI2)	−2.12	−2.05	1.62
Heli065300_st/HuBU027649/HanXRQChr10g0279571	AT3G29575	ABI five binding protein 3 (AFP3)	−2.78	−3.00	2.07
**Major CHO Metabolism**					
Heli015356_st/HuCL11595C001 HanXRQChr01g0001131	AT4G02280	sucrose synthase 3 (SUS3)	−2.60	−2.58	1.73
**Abiotic Stress Response**					
Heli007960_st/HuCL00349C002 HanXRQChr12g0376151	AT2G21620	Adenine nucleotide alpha hydrolases-like superfamily protein	−1.93	−1.72	1.94
Heli000924_st/HuCL00053C006 HanXRQChr15g0482351	AT3G50970	Dehydrin	−2.73	−2.64	1.75
**Oxidation-Reduction**					
Heli012215_st/HuCL05913C001 HanXRQChr03g0086571	AT4G11600	glutathione peroxidase 6 (GPX6)	−2.44	−2.51	1.75
Heli031078_st/HuDY913814/HanXRQChr13g0397121	AT2G27690	cytochrome P450	−2.18	−2.33	1.72
Heli042551_st/HuBQ968925/HanXRQChr13g0397121	AT2G27690	cytochrome P450	−2.35	−2.18	2.23
**Biodegradation of Xenobiotics**					
Heli001427_st/HuCL16610C001 HanXRQChr09g0274951	AT1G80160	lactoylglutathione lyase	−2.34	−1.88	1.69
**RNA Regulation of Transcription**					
Heli050332_st/HuBQ977024/HanXRQChr04g0116961	AT1G58330	transcription factor-related	−2.38	−2.82	2.25
**Protein Metabolism**					
Heli008339_st/HuCX945193/HanXRQChr01g0021981	AT3G04240	Tetratricopeptide repeat (TPR)-like superfamily protein	−2.04	−1.77	1.64
**Calcium Signalling**					
Heli003464_st/HuCL01023C003 HanXRQChr03g0079641	AT5G04220	Calcium-dependent lipid-binding (CaLB domain) family protein	−3.00	−2.42	1.61
**Development**					
Heli013690_st/HuCL04485C001 HanXRQChr13g0404461	AT3G22640	cupin family protein (PAP85)	−2.56	−2.70	1.69
Heli072838_st/HuBQ976615/HanXRQChr13g0404461	AT3G22640	cupin family protein (PAP85)	−2.83	−3.14	2.03
Heli010576_st/HuCL17138C001 HanXRQChr02g0043531	AT1G32560	Late embryogenesis abundant (LEA) group 1	−2.04	−1.77	1.88
Heli021725_st/HuCL11228C002/HanXRQChr04g0124391	AT5G44310	late embryogenesis abundant domain-containing protein	−2.57	−2.46	2.02
**Miscellaneous**					
Heli028250_st/HuCL13942C001 HanXRQChr14g0441431	AT2G26740	soluble epoxide hydrolase	−2.32	−2.59	1.74
**Transport**					
Heli011924_st/HuCL10648C001 HanXRQChr11g0342361	AT1G04560	AWPM-19-like family protein	−2.70	−2.60	2.06
**Not Assigned**					
Heli044202_st/HuCL15496C001 HanXRQChr17g0549071	AT4G12130	aminomethyltransferase activity	−2.42	−2.34	1.72
Heli019775_st/HuCL13794C001 HanXRQChr01g0000891	AT1G02700	Protein of unknown function	−2.65	−2.73	1.82
Heli026960_st/HuCL00001C521 HanXRQChr03g0071451	AT4G36700	Cupin, RmlC-type	−3.55	−3.15	2.23
Heli048093_x_st/HuCL00001C248	AT4G36700	RmlC-like cupins superfamily protein	−2.76	−2.19	1.64
Heli050559_st/HuBU023436/HanXRQChr05g0160341	no homologue	Uncharacterized protein	−2.68	−3.08	1.71
Heli052786_st/HuBU015421/HanXRQChr17g0545881	AT5G04010	Cyclin-like F-box	−2.76	−2.30	1.84
Heli059991_st/HuCL17581C001 HanXRQChr03g0084051	AT5G46640.1	Putative PPC domain	−2.32	−2.31	1.96
Heli060735_x_st/HuDY912393	no homologue		−1.70	−1.63	1.67
Heli068143_st/HuBU022800/	no homologue		−2.82	−2.71	2.39
Heli070447_st/HuDY931103/HanXRQChr06g0170581	no homologue	Uncharacterized protein	−1.58	−1.41	1.58

## Supplementary Materials

Supplementary materials can be found at http://www.mdpi.com/1422-0067/19/8/2464/s1.
