# Supplementary material for: One Way to Achieve Germination: Common Molecular Mechanism Induced by Ethylene and After-Ripening in Sunflower Seeds
Source: Int J Mol Sci. 2018 Aug 20;19(8):2464. doi: 10.3390/ijms19082464 (PMC6121958; doi:10.3390/ijms19082464)
Supplement: Supplementary file 1 [file ijms-19-02464-s001.zip › supplementary/Supplemental Figure S1.pptx]

## Slide 1
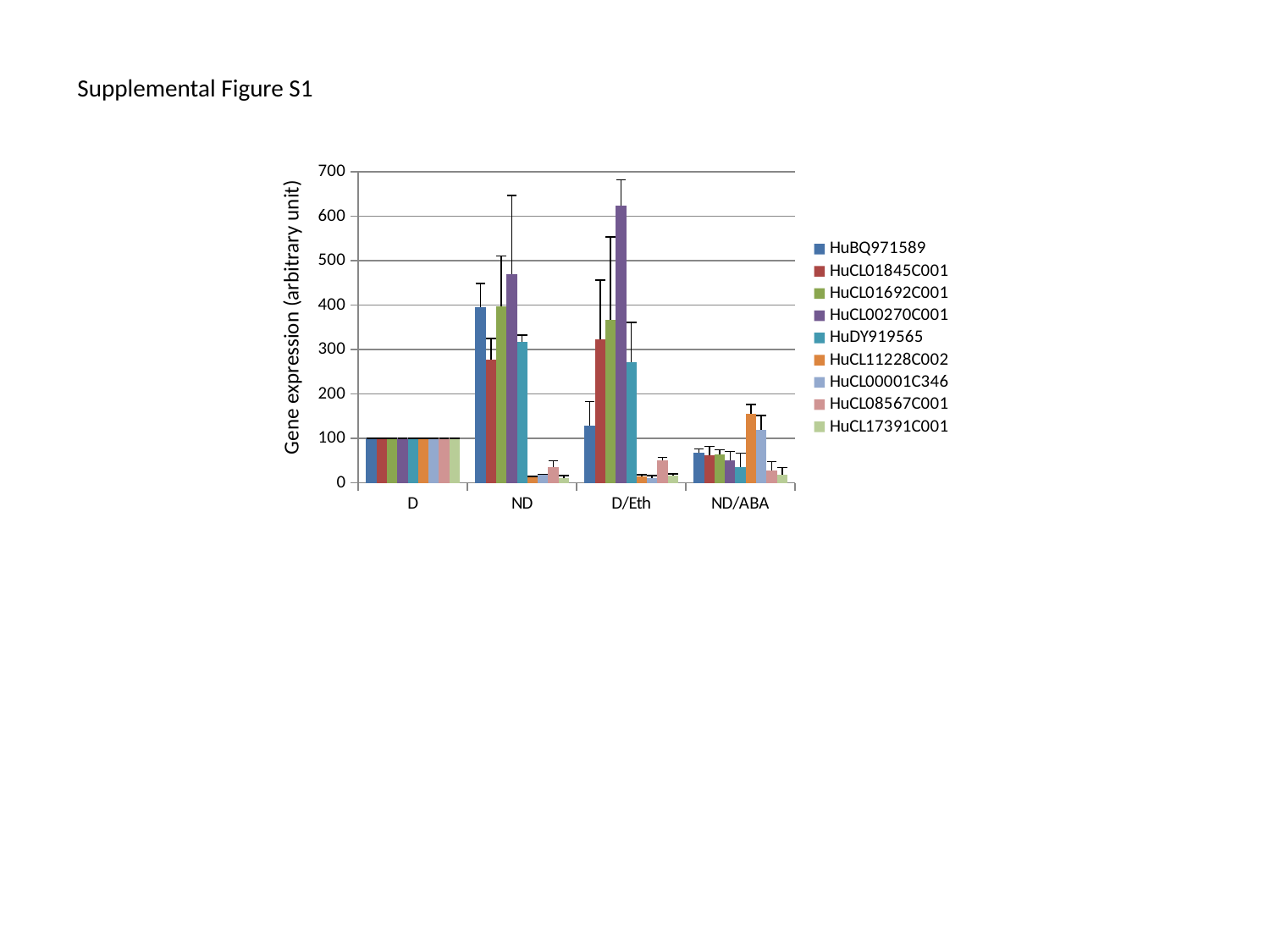

Supplemental Figure S1
### Chart
| Category | HuBQ971589 | HuCL01845C001 | HuCL01692C001 | HuCL00270C001 | HuDY919565 | HuCL11228C002 | HuCL00001C346 | HuCL08567C001 | HuCL17391C001 |
|---|---|---|---|---|---|---|---|---|---|
| D | 100.0 | 100.0 | 100.0 | 100.0 | 100.0 | 100.0 | 100.0 | 100.0 | 100.0 |
| ND | 395.3533333333333 | 277.1975 | 397.9525 | 470.7833333333333 | 317.3433333333333 | 11.855 | 17.5525 | 36.23666666666667 | 10.236666666666666 |
| D/Eth | 129.70000000000002 | 322.65749999999997 | 366.6675 | 623.86 | 271.79 | 13.855 | 11.115000000000002 | 49.9 | 17.206666666666667 |
| ND/ABA | 67.87333333333333 | 61.438 | 64.585 | 51.72666666666667 | 36.50666666666667 | 156.1425 | 118.92000000000002 | 28.21666666666667 | 18.616666666666664 |Gene expression (arbitrary unit)
